# Assessing Barriers and Difficulties to Healthcare Access Among Syrian Refugees in Jordan: An Observational Study

**DOI:** 10.3390/healthcare12222276

**Published:** 2024-11-14

**Authors:** Yazid Mohammed Gougazeh, Mahmoud Ola AlHussami, Konstantinos Tsaras, Wafa Hamad Almegewly, Savvato Karavasileiadou, Christos Kleisiaris

**Affiliations:** 1 Department of Community Health Nursing, Princess Muna College of Nursing, Mutah University, Karak 61710, Jordan; yazeedphdg@yahoo.com; 2 School of Nursing, University of Jordan, Amman 11942, Jordan; m.alhussami@ju.edu.jo; 3 Department of Nursing, School of Health Science, University Thessaly, Gaiopolis, 41500 Larissa, Greece; ktsa@uth.gr (K.T.); kleisiaris@uth.gr (C.K.); 4 Department of Community and Psychiatric Mental Health Nursing, College of Nursing, Princess Nourah bint Abdulrahman University, P.O. Box 84428, Riyadh 11671, Saudi Arabia; skaravasileiadou@pnu.edu.sa

**Keywords:** health determinants, healthcare services, Syrian refugees, accessibility, barriers to healthcare

## Abstract

(1) Background: Worldwide, refugees may have some difficulties in accessing healthcare services. However, little is known about the factors that may predict the level of accessibility to the public healthcare system in the host countries. (2) Aim: To examine the level of accessibility of Syrian refugees to the public healthcare system in Jordan and further to identify the prediction of socioeconomic factors and barriers to healthcare access. (3) Methods: A cross-sectional study was conducted with a convenient sample of 356 Syrian refugees residing outside camps (Irbid, Ajloun, and Jarash). Data were collected using the Access to Healthcare Services Scale instrument (adopted from the Canadian Community Health Survey), which is composed of 2 sections: the general access scale (8 items) and the difficulties scale (20 items). One-way ANOVA test and independent *t*-test were used to examine epidemiological correlations among variables, whereas a hierarchical linear regression model was used to examine the predictability of socioeconomic factors and barriers to overall access to the public healthcare system by exploring the incremental impact of additional predictors. (4) Results: the mean age of the 356 participants was 35.22 years old, 56.5% were female, 67.4% were married, most of them 46.1% have secondary education, and non-employed 69.9%. Significant associations were observed among participants with different marital status (*p* < 0.001), educational level (mean 11.85 vs. 19.85, *p* < 0.001), working status (15.47 vs. 17.93, *p* < 0.001), family household number (16.42 vs. 17.0, *p* < 0.001), and health insurance (none: 15.50 vs. governmental 24.50, *p* < 0.001). Multivariate analysis revealed that the most important factors that may predict the overall access to healthcare services were: family monthly income (beta −0.19, *p* < 0.001), household family number (beta 0.17, <0.001), health insurance (beta −0.09, *p* = 0.047), and barriers (beta −0.43, <0.001), even after adjusting for potential confounding effects: sex, age, educational level, and place of residence. (5) Conclusions: Our findings indicate that socioeconomic factors and barriers may considerably predict overall access to public healthcare in Jordan. It is crucially important, therefore, for the Jordanian government and international organizations to create and develop strategic plans and programs that enhance refugees’ access to health services, positively impacting their health and wellness.

## 1. Introduction

Since the beginning of the Syrian crisis in 2011, Syrian refugees in Jordan have been experiencing many difficulties in accessing healthcare. Such challenges include the lack of health insurance, elevated health service costs, and the fragmented healthcare system [1]. However, they reported some barriers to healthcare worldwide as geographical location, financial barriers, decreased level of awareness, and lack of employment [2,3,4,5]. Other barriers include some legal restrictions (i.e., refugee status, registration status, approvals for residence and employment), minimal work safety, poor job conditions (i.e., jobs with low-security levels, low payment, or temporary jobs without sustainability), and discrimination [6]. Furthermore, inadequate health insurance coverage, transportation difficulties, inability to access needed medications, lengthy waiting time, lack of reliable and valid information, and lack of formal and legal documentation of marriage, divorce, birth, or death cases were included in the list of barriers [7,8]. Additional barriers related to foreign cultures, such as language and communication barriers, were reported due to the lack of interpretation services [9].

In Jordan, healthcare services are delivered through the public/semi-public, the private sector, and the international non-profit health sector [10,11]. It is divided into four sectors: public sector (governmental and royal medical services), private sector, profit and nonprofit, and affiliated university hospitals and non-governmental organizations (NGOs). The principal healthcare provider in Jordan is the public health sector, which includes three major health sectors: the Ministry of Health (MOH), the Military Royal Medical Services (RMS), and two university-affiliated hospitals (Jordan University Hospital and King Abdullah University Hospital), which are interconnected via an unstructured referral system (Jordan National Health Account, 2021). The Ministry of Health delivers healthcare services to about 60% of the Jordan population, including the public governmental sector employees and their legally included relatives [2,11]. The healthcare services of the MOH are included in the civil health insurance program, which is paid for by the Ministry of Finance and the Ministry of Social Development in Jordan and financed by taxes and revenues. The healthcare services provided by the MOH are widely spread within Jordan and access most Jordanian and non-Jordanian populations, with particular concern for the less fortunate people, such as elders, children, women, people in rural and remote areas, and the non-Jordanian vulnerable people as refugees [12,13].

Syrian refugees were initially covered by MOH health services from 2011 to 2016, and this served as a heavy burden on the Jordanian health system and forced Jordan to ask for help from international organizations. Consequently, the United States Agency for International Development (USAID) responded by granting USD 85 million to support Jordan in continuing to provide healthcare services to Syrian refugees; after that, Syrian refugees, particularly those who reside within camps, receive free healthcare services supported by the United Nations High Commissioner for Refugees (UNHCR) and other charities based on a vulnerability assessment scope [14]. Through this period, co-payments were not needed for most primary health services, and care at public hospitals was accessible to refugees with referrals from governmental health centers. Whereas refugees presently have access to Jordanian public health facilities, and many endeavors are conducted to enhance considerably equal access to health services among refugees and the Jordanian population, they are currently needed to make co-payments equivalent to those required from the uninsured people in Jordan. Despite the financial support granted by the NGOs and UNHCR, healthcare costs for refugees are still the most apparent barrier to accessing healthcare [15].

Based on the above, Syrian refugees experience many barriers that prevent or decrease their access to healthcare services in the host countries, and this may force them to ignore their health problems, delay the utilization of healthcare services, or force them to adopt unhealthy practices, which may affect their health negatively [16,17,18,19]. As Syrian refugees are scattered in the Jordanian communities and utilize the Jordanian public health sector, they may affect the public health of the Jordanian people because of the heavy demands on the Jordanian health system (services, institutions, personnel, and supplies) made by Syrian refugees, which may cause additional burden on it, which may lead to weakening the health system and lowering its willingness to serve the Jordanian people, which could result in dissatisfied health services delivered and unwanted health outcomes. Therefore, this study was conducted to examine the level of healthcare accessibility by Syrian refugees in Jordan and further investigate whether socioeconomic factors and barriers may predict the accessibility to the public healthcare system in Jordan.

## 2. Materials and Methods

### 2.1. Design

A self-reported descriptive, correlational research design was used in this study. Data was collected from Syrian refugees who are residing outside the camps in the cities of Irbid, Ajloun, and Jerash, where many Syrian refugees resided since the beginning of the Syrian conflict. These cities are close to the Jordanian–Syrian borders and contain about 25% of the total Syrian refugees in Jordan [20]. The researcher obtained lists containing addresses and contacts of Syrian refugees in the selected areas, and the participants were openly invited to participate in the study.

### 2.2. Population and Eligibility

The target population was all Syrian refugees who were forced to leave their homes due to the Syrian conflict that started in 2011, crossing the Jordanian–Syrian borders and residing outside the camps in Jordan. The accessible population was all Syrian refugees living in the selected northern cities of Jordan who met the inclusion criteria of this study and were available in the settings chosen at the time of data collection.

The inclusion criteria included Syrian refugees who arrived in Jordan due to the Syrian conflict in 2011, reside outside the camps, are over 18 years old, and agree to participate. Exclusion criteria include refugees who are disabled, handicapped, and those who have mental illness.

### 2.3. Sample and Sampling

A convenient sample was used in this study to recruit the sample; however, snowball sampling was also used, by which the early recruited Syrian refugees in the study will be asked to refer other refugees who met the inclusion criteria of this study. The researcher used the lists of refugees’ addresses and contacts to access the refugees; some guide people (i.e., religious men and charity people) were also asked to access refugees. It is not simple to find refugees because they are scattered among the Jordanian population and frequently transfer from one place to another. Hence, the researcher used most participants as reference points to refer to others. Linear multiple regression: Fixed model, R2 deviation from zero power analysis was conducted using G*Power version 3.1.9.4 to estimate the sample size. To identify the required sample size, an error probability α of (0.05), a power of (1 − β) of (0.90), a small effect size of (0.07), and 12 predictors. The estimated sample size was 323; however, it was decided to add 10% to compensate for the expected missing data resulting from uncompleted and any possible withdrawal from the study. Accordingly, a total of 400 participants will be recruited for the study.

The study’s sample was recruited from these three governorates: (178) participants from Irbid, (178) participants from Jarash and Ajloun, and (89) participants from each (89 participants from Jarash governorate and 89 participants from Ajlune governorate). Based on the proportions of Syrian refugees in Jordanian cities, the Irbid governorate contains about 25% of the total Syrian refugees, whereas those in Jarash and Ajlune governorates are about 5%. Therefore, half of the sample was recruited from Irbid.

### 2.4. Measurement Instruments

A structured questionnaire with a transparent cover letter was used in this study. The questionnaire is composed of two sections: (1) socio-demographic data, and (2) the Access to Healthcare Services Scale, which is adapted from the Canadian Community Health Survey (CCHS 2.1) [21], which all were Likert scale questionnaires. The researcher selected only two sections from CCHS based on research questions, and then the validity and reliability tests were conducted. The two sections are of the General Access Scale and include two sections: the first concerns the access and utilization of outpatient clinics (four items). The second section comprises eight items representing overall access to healthcare. The other section of the healthcare access scale is the “Difficulties Scale” (20 items), measuring the barriers against refugees accessing health services. All scores of the General Access Scale items were summated for each participant to represent the accessibility variable and range from 8 to 40. A higher score indicates greater accessibility to the public healthcare system. On the other hand, all items were summated for each participant to estimate get the total score of difficulties variable (20 items), ranging from 20 to 100. Higher scores indicate greater perceptions of barriers and difficulties in accessing healthcare services.

Validity testing was performed, and the content validity index CVI for both access and difficulty scales was 98.3% and 95%, respectively. Concerning reliability measurement, the results revealed a Cronbach’s alpha of 0.78 and 0.85 for the access and difficulty scales, respectively. Culturally, the instrument was used in a study before this study was conducted with the same population (Syrian refugees). In addition, after conducting the pilot study, the researcher made some modifications to meet the cultural differences.

Based on the literature review, the researcher selected some variables, such as age, social status, educational level, working status, total monthly income, source of income, number of family households, presence of health insurance, and presence of barriers to access healthcare services, to examine their effect on the accessibility of healthcare services among Syrian refugees.

### 2.5. Data Collection

After obtaining the IRB from the University of Jordan, the researcher obtained permission from the Jordan Hashemite Charity Organization (JHCO) to collect data from Syrian refugees by using registration data regarding Syrian refugees, which contains lists of contact numbers and residency places. In addition, similar lists were obtained from the charitable associations that deal with Syrian refugees in the three cities. Firstly, the participants were asked if they were Syrian refugees who left their homes due to the Syrian conflict in 2011, and then the researcher explained the nature, the purpose, and the benefits of the study. A consent form was obtained for the participants who agreed to participate and met the inclusion criteria. The researcher was physically present with refugees when they filled out the questionnaire to answer any questions the participants may have; the time needed to answer the questionnaire will be about 10–20 min. Questionnaires were self-administered for the educated refugees, while the illiterate refugees completed the questionnaires through the structured interview technique.

Each questionnaire was coded with numbers from 1–356; after the participants completed the questionnaire, every 25 questionnaires were put in an envelope, and then the code numbers were written on these envelopes (to facilitate checking any questionnaires during the analysis). The data from the questionnaires were entered into the SPSS system daily after filling out the questionnaires. The hard copy of the questionnaires was kept in a safe and locked wardrobe. Only the researchers have access to the data. No one has access to the data. The data were collected over four months. Finally, a total of 356 out of 400 questionnaires that were initially distributed were identified with no missing data and involved in the statistical analysis (response rate 89%).

### 2.6. Ethical Consideration

Ethical approval was obtained from the IRB committee. Then, the approval is obtained personally from the refugees. The cover letter of the questionnaire is placed on the first page and includes the name and contact information of the researcher (researcher’s email), the purpose and nature of the study, and ensuring that the survey will not include any physical, psychological, or economic harm for them. In addition, anonymity and confidentiality were maintained (each questionnaire had a coded number of participants without names). In addition, participants can withdraw from the study at any time.

### 2.7. Statistical Analysis

Statistical Package for the Social Sciences (SPSS) package version 26 was used in the analysis, and many statistical tests were used based on the research questions. Descriptive statistics (mean, percentage, and standard deviation) were used to describe the study sample demographics. Other statistical tests, such as the one-way ANOVA test and independent *t*-test, were used to examine the differences in the mean scores of accesses according to the refugees’ demographical variables. Multivariate analysis, particularly the hierarchical linear regression model, was applied to examine the effect of barriers (Difficulties Scale score) and social and economic factors (predictor variables) on accessibility (B section of the General Access Scale-dependent variable). Sex, age, educational level, and place of residence were also examined and controlled for potential confounding effects (adjustment variables).

## 3. Results

### 3.1. Demographic Characteristics

Table 1 shows that 56.5% of participants were female, and the sample mean age was 35.22 (S.D. 14.04). However, 82.6% of the sample were of primary or secondary level, and 67.4% were married. Regarding employment status, 69.9% have no work.

The mean household monthly income was 209.78 (S.D. 84.81), and 60.4% of the sample’s income resource was from “a working family member”, 74.4% were living in rural areas, and 59.6% had more than three family members, while 32.3% had three family members. Moreover, 69.1% of the sample do not have any health insurance.

### 3.2. Access Public Healthcare System

The total score was calculated to determine the public healthcare system’s access level. Results revealed that the total scores of participants’ level of access to the public healthcare system ranged from 8 to 32, with a mean of 16.09. Notice that the higher the mean, the higher the level of access to the public healthcare system. The quartile equation revealed that 75% of the participants had a score of 13 or higher, and 50% had a score of 16 or higher. This indicates that participants had low access to the public healthcare system, in which the cutoff point was determined by a score of 20.

Table 2 revealed that the access item that has the highest agreement rate was “Your medical diagnosis and history are known for you and health care givers” with mean responses of 2.58, then “You receive adequate information about your health state and medications from the medical team.” with mean responses of 2.26. However, the access item that has the lowest agreement rate was “Always you can pay for the health services costs” with mean responses of 1.64, then “Always you can pay for the prescribed medications” with mean responses of 1.65. 

Table 3 shows that 26.4% of the sample had attended investigation services, 20.8% of the sample had attended medical services, and 20.2% of the sample had attended emergency services. In addition, it appears that none of the sample has a private car as transportation to the public healthcare system, while 68.8% of the sample use a bus as transportation to the public healthcare system. Regarding distance to the healthcare system, 51.1% of the sample describe the distance between their homes and healthcare centers as “close”, and 48.3% of the sample describe the distance between their homes and healthcare centers as “far”.

### 3.3. Barriers to Access to the Public Healthcare System

In Table 4, we present the level of barriers to access to the public healthcare system. Results revealed that the total scores of participants’ level of barriers to access to the public healthcare system ranged from 37 to 96 with a mean of 66.85. Note that the higher the mean, the higher the level of barriers to access to the public healthcare system. The quartile equation revealed that 75% of the participants had a score of 61 or higher, and 25% of the participants had a score of 72 or higher. This indicates that participants had moderate to high levels of access to the public healthcare system. The most important barrier to access was “Long waiting time to take medications” (mean = 4.37) and then “Difficulty in affording treatment” (mean = 4.17). Yet, the barrier to access item that has the lowest agreement rate was “Inability to leave the house due to health problems” (mean = 1.85), then “Difficulty to access health service due to very bad health status” (mean = 1.90).

### 3.4. Differences Among Socioeconomic Variables with Respect to Accessibility

Table 5 shows the associations of socioeconomic variables with accessibility. Particularly, the analysis of variance showed significant differences as regards accessibility to the public healthcare system were observed in participants with differential: marital status (*p* < 0.001), educational level (mean 11.85 vs. 19.85, *p* < 0.001), working status (15.47 vs. 17.93, *p* < 0.001), family household number (16.42 vs. 17.0, *p* < 0.001), and health insurance (none: 15.50 vs. governmental 24.50, *p* < 0.001).

### 3.5. Factors Predicting Overall Access to the Public Healthcare System

In Table 6, the effect of socioeconomic factors and barriers as predictive variables on overall access to healthcare is presented. Specifically, families with <4 members had greater accessibility (beta 0.17, <0.001) to the public healthcare system compared to families with 4 or more members. Also, participants with a family monthly income of less than 210 € (beta −0.19, *p* < 0.001) and those having no health insurance (beta −0.09, *p* = 0.047) had decreased accessibility in comparison to those with the highest monthly income and health insurance, respectively. Likewise, facing more barriers in daily life was also associated with decreased accessibility (beta −0.43, <0.001) to the public healthcare system, suggesting that fewer barriers have more accessibility, even after adjusting for potential confounding effects (age, sex, educational level, and residence).

## 4. Discussion

This study examined the difficulties in accessing healthcare services faced by Syrian refugees in Jordan and further identified the most important socioeconomic factors that may predict the accessibility of the public healthcare system. Data analyses revealed that having health insurance, increased monthly income, fewer family household members, and facing fewer barriers (20 items) were the most important predictors for greater healthcare accessibility. Moreover, participants with variance in marital status, educational level, working status, family household number and health insurance experienced significantly diverse accessibility.

The main findings of the present study were that accessibility to the public healthcare system in Jordan is significantly affected by socioeconomic factors and barriers such as health insurance and monthly income, including household family members, suggesting that fewer difficulties experienced by refugees in daily life are associated with greater accessibility to the public healthcare system. Possible explanations for these associations could be that refugees facing housing instability and financial struggles may prioritize basic needs over healthcare, which could further limit their access to public healthcare [18,22]. In addition, most refugees were females; this might be due to accessibility limitations as the time chosen to meet refugees was in the morning when most male members were out at work or searching for jobs. Moreover, most refugees were married, which might be because Syrian culture encourages early marriage [23,24]. However, most participants were unemployed due to the lack of job opportunities and some legal restrictions regarding employment in Jordan [25]. Even so, most participants had more than three household members, which might be related to the Islamic and Arabic culture, which encourages reproduction. Additionally, most refugees had primary or secondary educational levels and lived in rural areas, and this might be interpreted as due to low economic status and challenging living circumstances. This was highly consistent with the results of a study conducted in Turkey [26].

Commonly, most participants had low total income, mainly from the jobs of some family members or NGO aids, and they were dissatisfied with their incomes. This might be justified as most Syrian refugees are of low economic status because they left their homes, properties, jobs, and financial resources in Syria and are suffering from additional economic crises due to a lack of employment, difficult living conditions, and lack of pocket money. All these findings were consistent with the findings of reports and studies conducted concerning the living conditions of Syrian refugees in some hosting countries [25,26,27]. Even the Jordanian population is suffering from similar complaints; that is, low job opportunities are mainly responsible for the low socioeconomic status of most Jordanians [28].

We also found significant differences among levels of education toward accessibility to healthcare services. However, this association was not significant after adjusting for confounding effects (model 2), suggesting that educational level was not a significant predictor for increased and/or decreased access to the public healthcare system of Jordan. In comparison to our findings, recent studies conducted in neighborhood countries reported that educated persons commonly possess good knowledge regarding health issues and an accepted level of health literacy, which force them to adopt healthy attitudes and behaviors and demonstrate a higher level of access and utilization of the healthcare service. Findings were consistent with studies conducted in Lebanon and Turkey [19,28]. However, these studies performed bivariate analyses and not adjusted regression models to explore this association.

Importantly, participants with a family monthly income of less than 210 € had decreased accessibility in comparison to those with the highest monthly income. This might be justified as total income represented the actual financial status of the refugees; that is, when they have adequate income, they can access healthcare services more efficiently because this enables them to pay for health services, including examinations, admissions, medications, referrals, surgeries, and transportations. Findings were congruent with those of several studies conducted on Syrian refugees worldwide [19,27,28]. Likewise, the source of income seems to be another significant factor, as refugees who depend on NGOs have more access to healthcare services than those who rely on other income sources. A possible justification for that is that most Syrian refugees depend on NGOs to get their income because it is regular monthly, which enables them to access health services sufficiently. This was consistent with the results of two studies conducted in Jordan [29,30]. Similarly, the number of household members was reported as a significant factor. The possible explanation for that could be that the families of large numbers of households experienced more financial burden because most families have low economic status and strive to maintain the basics for their lives, such as housing, food requirements, education, and health; therefore, sometimes they found themselves unable to access healthcare services due to the lack of pocket money.

Most importantly, this study showed that Syrian refugees having no health insurance reported significantly decreased accessibility in comparison to those with any type of health insurance. This might be explained by the fact that this study’s sample targeted refugees who resided outside the camps and who mostly missed health insurance when they left the camps. Further, those who have health insurance mostly obtain it from NGOs. These results were congruent with those of a study conducted in Lebanon. Likewise, health insurance is another significant factor affecting access to health services. This is not surprising because it is considered one of the fundamental requirements for accessing healthcare services. Many studies conducted regarding Syrian refugees worldwide indicated the same results [28,31,32].

The most rated barriers (difficulty items) to access reported in this study were “Long waiting time to take medications”, and “Difficulty in affording treatment”. However, the lowest agreement rated were “Inability to leave the house due to health problems” and “Difficulty to access health service due to very bad health status”. Indeed, this is not strange because refugees who experienced more barriers to healthcare services would not be able to access and utilize healthcare services, whatever the barriers. This was consistent with most studies conducted regarding access to healthcare services among Syrian refugees [27,28,32]. In more detail, most Syrian refugees have some barriers hindering them from accessing healthcare services; however, some refugees have access to healthcare services, but they do not prefer to utilize them due to some reasons such as lack of awareness regarding health-related issues, tending to use complementary medicine (herbs), and considering the health problems as issues without priority, which all at the end will worsen their health states.

Transportation difficulties were also reported in our study. Presumably, most refugees implied that the bus was the primary vehicle used in accessing health services; this is not surprising because, according to legal regulations, Syrian refugees cannot possess their cars with Jordanian labels, in addition to their financial inability to do so. Even though the bus is considered the most used vehicle among all people, even Jordanians, because it is the cheapest and most available, most participants described healthcare services as closed, and this might be because most refugees reside in rural areas where health centers are closed to all people, and due to a lack of crowding and traffic problems, which could facilitate access to health centers easily. However, these findings were inconsistent with a study investigating the possible barriers to accessing healthcare among Syrian refugees in Canada [33]. Specifically, the access item with the highest agreement rate was “Your medical diagnosis and history are known for you and health caregivers”, which is unsurprising. Typically, most refugees and caregivers are aware of participants’ health problems; that is, participants need to know about their health conditions to look after themselves, and the health workers should know how to provide the appropriate care for them. However, the access item with the lowest agreement rate was “Always you can pay for the health services costs”. This might be interpreted based on the refugees’ poor financial status and lack of health insurance to cover payment requirements. These findings were consistent with those of many studies conducted around the world [34,35]

Otherwise, the results indicated that participants had moderate to high levels of barriers to public healthcare services, in which the barrier to access items with the highest agreement rate was “Long waiting time to take medications”, then the item of “Difficulty in affording treatment”. Yet the barrier to accessing the item with the lowest agreement rate was the “Inability to leave the house due to health problems”. A possible justification for that might be that waiting time is considered the significant barrier to accessing healthcare, particularly to getting medications [35], because dispensing medications mainly includes some procedures prone to delay the process of dispensing, including preparing medications, payment, and referral to a specialist in case of drug unavailability. Also, the poor financial status of most participants is considered the primary reason for the barrier of the difficulty to afford treatment. The lowest barrier was the inability to leave the house due to health problems; this might be due to most participants being young and not having fatal diseases that prevent them from mobility, in addition to the low prevalence rate of chronic diseases. These findings were similar to those of some studies conducted to investigate the barriers to health services among Syrian refugees [10,31].

### 4.1. Future Implications

The current study provides clear information for policy developers and researchers to develop a holistic strategy to enhance Syrian refugees’ access to health services in Jordan [5]. Nurses could design creative educational programs that are tailored toward low-income, illiterate, and unemployed Syrian refugees to improve their access to healthcare services. Nurses could provide counseling and referral services, connect Syrian refugees with social bodies, and provide financial, social, and psychological support. Also, nurses can play an important advocacy role in connecting refugees’ voices to the formal administrators to stand on their needs regarding health, employment, resettlement, and finance. Further quantitative studies with large and multi-setting samples should be conducted, in addition to conducting qualitative studies to explore the actual lived experiences of Syrian refugees regarding access to health services and barriers that hinder such access.

### 4.2. Limitations and Strengths

Although this study has many strengths, such as recruiting a large sample, using valid and reliable instruments, and being conducted in multiple sites, it has some limitations. For example, it was performed in specific cities in Jordan. It did not include Syrian refugees from all Jordanian towns, and it used a convenient sample, which may limit the study results’ generalizability. In addition, the study was cross-sectional. Therefore, the causal relationship between social determinants or barriers to access to health services cannot be ascertained. It was preceded by a pilot study, comprised of a relatively large sample size based on widely valid, reliable, and standardized instruments in data collection and the multi-setting property, as it was conducted in three different cities in the north of Jordan. However, the study surveyed the participants, which enriched the researchers’ knowledge of the continuum of healthcare services they follow and further shed light on another area of significance to the study.

On the other hand, some limitations of this study were present. Firstly, most participants were females because the researcher filled out the questionnaires in the morning when most male refugees were out working or searching for jobs, and only females were available to complete the questionnaires. Secondly, although the study was conducted in three settings in Jordan, the generalizability might be limited because the three settings are located in one province in Jordan (the northern), which has, to some extent, many properties distinguishing it from other provinces in Jordan, which in turn may influence the results of the study. In other words, we cannot generalize the results of these studies to the Syrian refugees who reside in Amman or the southern cities because these settings have different properties and circumstances.

Thirdly, because this study used a cross-sectional design, causal relationships between dependent and independent variables cannot be ascertained. Fourthly, the study sample was convenient, which may lead to selection bias, limiting the generalizability of the study results. Otherwise, self-report questionnaires may lead to misinterpretation in answering the questions by the participants and the threat of recall bias and socially desirable answers.

## 5. Conclusions

This study revealed that Syrian refugees having no health insurance, “poor” family monthly income, increased household family members, and facing several barriers and/or difficulties were presented with decreased accessibility to the public healthcare system in Jordan. These findings indicate that socioeconomic factors and barriers may considerably predict the overall access to healthcare. Thus, the Jordanian government and international organizations should pay more attention to Syrian refugees to overcome the barriers to accessing healthcare services, enhance their access to health services, maintain and improve their health, and protect them from health problems. It is crucially important, therefore, that local health policies are developed and supported by global bodies. Such policies should focus on providing Syrian refugees with comprehensive health insurance, conducting national campaigns and outreach programs to access refugees in rural areas, expanding awareness activities, and activating the referral and follow-up programs.

## Figures and Tables

**Table 1:** Socio-demographic data (*n* = 356).

Categorical Variables			
Variables		*n*	%
Gender	Male	155	43.5
	Female	201	56.5
Marital Status	Single	73	20.5
	Married	240	67.4
	Divorced	17	4.8
	Widowed	26	7.3
Education level	Illiterate	7	2.0
	Primary	130	36.5
	Secondary	164	46.1
	Diploma	34	9.6
	Bachelor	21	5.9
Employment	None	249	69.9
	Agriculture	23	6.5
	Industry	21	5.9
	Construction	17	4.8
	Trade	32	9.0
	Others	14	3.9
Income sources	NGOS	90	25.3
	Working family member	215	60.4
	Relatives or friends	51	14.3
Residence	Urban	91	25.6
	Rural	265	74.4
Household no.	One	7	2.0
	Two	22	6.2
	Three	115	32.3
	More than three	212	59.6
Health Insurance	None	246	69.1
	Governmental	0	0
	NGOS	79	22.1
	Private	31	8.7
Continuous Variables			
Age		35.22	14.04
Monthly income		209.78	84.81

**Table 2:** Overall access to the public healthcare system (*n* = 356).

Items	M	SD
You can obtain the desired health service when you need	2.17	0.84
You can access the specialist at any time	2.04	0.71
You can always pay for the health services costs	1.64	0.66
You can always pay for the prescribed medications	1.65	0.70
Always you can get the prescribed medications	1.86	0.74
Your medical diagnosis and history are known to you, and for health care givers	2.58	1.00
You receive adequate information about your health state and medications from the medical team	2.26	0.86
You have a regular caregiver to access when needed	1.89	0.67

Abbreviations: M: mean value; SD: standard deviation.

**Table 3:** Access and utilization of outpatient clinics to the public healthcare system (*n* = 356).

Items		*n*	%
Attended health services	Medical	74	20.8
	Surgical	35	9.8
	Investigations	94	26.4
	Health education	28	7.9
	Emergency	72	20.2
	other	53	14.9
Type of transportation used	Walking	20	5.6
	Taxi	80	22.5
	Bus	245	68.8
	Private car	0	0
	Car of a friend or relative	11	3.1
Distance of health center	Very close	31	8.7
	Close	151	42.4
	Far	166	46.6
	Very far	6	1.7
	I don’t know	2	0.6
Time you need to reach the health service	Less than 15 min	51	14.3
	16–30 min	149	41.9
	31–60 min	147	41.3
	More than 60 min	7	2.0
	I don’t know	2	0.6

**Table 4:** Barriers and difficulties to access to the public healthcare system (*n* = 356).

Items	M	SD
Difficulty in obtaining a referral to receive treatment in other health agencies	3.67	0.77
Difficulty in obtaining an appointment in the needed clinic	3.74	0.73
Long waiting time between getting an appointment and the visit	3.85	0.70
Long waiting time to see the specialist in the clinic	3.90	0.69
Lack of the health service you need in the health center you attend	3.12	0.80
Lack of the health service you need in your residence place	3.01	0.85
Difficulty in transportation to reach the health service	3.58	0.82
Difficulty in affording treatment	4.17	0.66
Lack of money for transportation	4.01	0.80
Difficulty accessing health services due to terrible health status	1.90	0.92
Change or cancelation of appointment	3.03	1.12
Inability to leave the house due to health problems	1.85	0.90
Difficulty in obtaining a medical diagnosis or diagnostic investigations	3.16	0.97
Difficulty in communication with physicians	3.35	0.81
Difficulty in communication with nurses	3.30	0.83
Inadequate information or medical advice from the health team	3.13	0.89
You don’t know, or no one tells you where to go to receive health service	3.01	0.97
Difficulty in obtaining health service during official working hours	2.84	0.85
Difficulty in obtaining health service outside the official working hours	3.87	0.68
Long waiting time to take medications	4.37	0.66

Abbreviations: M: mean value; SD: standard deviation.

**Table 5:** Associations of socio-demographic variables with accessibility (*n* = 356).

Categorical Variables	Mean ± SD	F	*p*-Value
Marital status		8.353	<0.001
Single	17.23 ± 3.35		
Married	16.20 ± 4.44		
Divorced	14.76 ± 3.28		
Widowed	12.69 ± 3.53		
Educational level		12.803	<0.001
Illiterate	11.85 ± 3.93		
Primary	15.20 ± 3.75		
Secondary	15.89 ± 3.83		
Diploma	19 ± 5.48		
Bachelor	19.85 ± 4.09		
Working status		4.814	<0.001
None	15.47 ±3.96		
Agriculture	16.60 ±4.12		
Industry	18.04 ±4.18		
Construction	16.05 ±3.92		
Trade	17.93 ±5.69		
Others	19.07 ±3.38		
Source of income		12.510	<0.001
NGOs	14.27 ± 3.46		
Working family member	16.86 ± 4.43		
Relatives or friends	16 ± 3.87		
Family household number		7.160	<0.001
One	16.42 ± 1.81		
Two	18.68 ± 4.76		
Three	17 ± 4.21		
Four or more	15.31 ± 4.10		
Health insurance		13.131	<0.001
None	15.50 ± 3.66		
Governmental	24.50 ± 3.53		
NGOs	16.25 ± 4.97		
Private	19.77 ± 4.54		

Abbreviations: M: Mean value; SD: standard deviation. Notes: Methods: one-way analysis of variance (ANOVA) for categorical variables.

**Table 6:** Effect of socioeconomic factors and barriers (20 items) on overall access (8 items) to healthcare.

	Unadjusted (Model 1)					Adjusted (Model 2)				
	*β*	*SE*	Beta	*sr*	*p*-Value	*β*	*SE*	Beta	*sr*	*p*-Value
Intercept (*α*)	30.93	1.46			<0.001	30.71	1.49			<0.001
Social Factors										
Marital Status (not married vs. married)	−0.24	0.41	−0.03	−0.03	0.559	−0.25	0.43	−0.03	−0.03	0.559
Household (<4 vs. ≥4)	1.50	0.40	0.17	0.17	<0.001	1.45	0.40	0.17	0.16	<0.001
Economic Factors										
Health Insurance (no vs. yes)	−0.83	0.42	−0.09	−0.09	0.046	−0.83	0.42	−0.09	−0.09	0.047
Monthly Income (<EUR 210 vs. ≥EUR 210)	−1.56	0.40	−0.18	−0.17	<0.001	−1.66	0.42	−0.19	−0.17	<0.001
Difficulties Scale score										
Barriers (20 items)	−0.21	0.02	−0.44	−0.42	<0.001	−0.21	0.02	−0.43	−0.39	<0.001
Adjustment Variables										
Sex (male vs. female)						−0.29	0.40	−0.03	−0.03	0.472
Age (years)						−0.01	0.02	−0.01	−0.01	0.860
Education (primary vs. secondary or higher)						−0.47	0.42	−0.05	−0.05	0.259
Place of Residence (rural vs. urban)						0.72	0.44	0.07	0.07	0.102

Abbreviations: *SE*: standard error; *sr*: semi-partial correlation coefficient. Notes: Methods: multivariate analysis, hierarchical linear regression model; general access scale score placed as outcome variable; social factors, economic factors, and difficulties scale score (barriers) as predictor variables; sex, age, educational level, and place of residence as adjustment variables. Model 1: F = 33.460, *p* < 0.001; *R*^2^ = 0.323; adjusted *R*^2^ = 0.314. Model 2: F = 19.110, *p* < 0.001; *R*^2^ = 0.332; adjusted *R*^2^ = 0.315.

## Data Availability

Data are contained within the article.
